# Use of an automated feedback application to improve communication skills

**DOI:** 10.15694/mep.2018.0000011.1

**Published:** 2018-01-15

**Authors:** Kwang Meng Cham, Jia Jia Lek, Jeremiah K. H. Lim, Anthea L. Cochrane

**Affiliations:** 1 University of Melbourne

**Keywords:** technology-enhanced learning, communication skills, mobile learning, pedagogical strategies, optometry

## Abstract

This article was migrated. The article was marked as recommended.

Effective communication skills are a professional competency, yet are often overlooked during training. Providing immediate and constructive feedback is imperative to assist students in developing better communication skills. We sought to evaluate the educational value of using a university-developed application, *Rapid Feedback*, to provide feedback following students’ oral presentations over two years.

An online survey comprising of eight 5-point Likert scale items and one open-ended question was conducted in 114 (response rate = 86.5%) students. Students either strongly agreed or agreed that the feedback delivered was timely (98%), relevant (96%), high quality (90%), and specific to enhance their learning (87%). The feedback obtained has helped to identify strengths and weaknesses (87%). Students commented that feedback received will improve their communication skills (90%). The report was also shown to supplement verbal feedback (95%). Overall, students expressed that the feedback report was valuable, allowing for critical self-reflection and future retention. Staff have also found the application easy to use and administer.

In a time- and resource-constrained teaching environment, educators constantly explore technology to support student learning and teaching outcomes. We have implemented an application that is user-friendly to staff, efficient, and has provided effective feedback that is well-received and valued by students.

## Introduction

Effective communication skills are a core generic attribute of allied health professionals ( Heisler et al. 2002; Bredart et al. 2005; Kafetsios et al. 2014; Kiely et al. 2015). Traditionally, most teaching time has been spent on acquiring knowledge and mastering clinical skills. Less attention has been focused on developing other general attributes such as communication skills. Educators are also faced with the challenge of providing specific and timely feedback when teaching large classes due to time constraints. Here, we sought to evaluate the educational value of using a university-developed digital application *, Rapid Feedback*, to provide automated feedback following oral presentations.

## Methods

The *Rapid Feedback* application allows an assessor to deliver real time formative and/or summative assessment and feedback of a student’s oral presentation. The criteria include voice, pace and confidence, presentation structure, quality of slides/visual aids, knowledge of material, content and concluding remarks. As the assessor grades the students, each mark is tagged with a pre-written feedback paragraph. Different versions of pre-written comments are embedded within each criterion to ensure all students do not receive the same comments. Upon completion, all selected feedback is compiled, and a structured individualized feedback report is generated that can be e-mailed to students instantly. The application has the flexibility to add and edit comments, and allows for audio-recorded feedback. Students’ grades are automatically collated in an excel spreadsheet and sent directly to the subject coordinator.

To assess its effectiveness in the classroom, we used this application to provide feedback to Year 1 students over a two-year period following group oral presentations. Each group had six students to work on a clinical case scenario. All students were aware of the assessment rubrics, and knew that they would receive standard verbal feedback. In addition, a personalized feedback report would also be sent to them at the end of their presentation. A week after receiving the feedback report, students completed an online anonymous survey assessing feedback quality and application satisfaction. The survey was conducted with prior ethics approval (ID: 1443441.1) at the University of Melbourne. It comprised of eight 5-point Likert scale items and an open-ended question. For each item, students were presented with a statement and asked about the extent to which they agreed (5- Strongly Agree, 4-Agree, 3-Neutral, 2-Disagree, 1-Strongly disagree).

## Results

The survey was sent out to a total of 132 students, of which 114 responded (86.5%). To test the reliability of the responses, a Cronbach’s alpha of 0.97 ± 0.01 was obtained, suggesting high internal consistency. Median scores ranged between 4 and 5 for all the questions, indicating a positive impact on student learning and high satisfaction with the quality of feedback from the application. Indeed, most students either strongly agreed or agreed that the feedback report was timely (98%), relevant (96%), was of high quality (90%) and specific enough to improve on their learning (87%). Students felt that the feedback obtained through this application not only helped to identify their strengths and weaknesses (87%), it also helped them to improve their communication skills (90%). As students could retain a copy of the report, most felt that this was an excellent supplement to the verbal feedback (95%) received. Students commented that the feedback report generated by the *Rapid Feedback* application was truly valuable, allowing for critical self-reflection and future retention. Staff (n=8) have also commented that the application was easy to use and administer. It significantly reduced workload by approximately 50% when collating marks, and written feedback was feasible, and could be delivered with little delay.

## Discussion

Skilled communication skills underpin effective practice and is a vital professional competence. Studies have shown that good communication skills can be learned ( Parry et al. 2009; Joyce et al. 2011; Moore et al. 2013), is beneficial in early stages of health professional courses ( Ali et al. 2017), and can increase patient satisfaction ( Heisle et al. 2002). But, without immediate and constructive feedback, which is critical to student learning, it can be challenging for students to attain this competency ( Weaver 2006; Hattie et al. 2007; McKimm 2009). The issue of feedback is further complicated by the type, format, timing, expert judgement, and maturity and life experience of the students ( Hattie et al. 2007; Murdoch-Eaton et al. 2012). There were also concerns regarding feedback quality and delivery being variable and contradictory ( Groves et al. 2015). In all, it highlights a need for a consistent approach to giving feedback to support student learning and teaching outcomes.

The *Rapid Feedback* application serves to deliver objective and consistent written feedback that is timely and specific. It helps the students to reflect on their performance, potentially enhancing personal growth and communication behaviors. The application is practical, reliable and easy to administer. Students have found the technology-enhanced learning experience beneficial, with improving communication skills coming through as a strong positive theme within both the narrative and quantitative feedback.

## Conclusion

Providing feedback can significantly impact on a learner’s acquisition and retention of skills ( Hattie et al. 2007). In a time- and resource-constrained teaching environment, we have implemented a digital automated feedback application that is efficient and user-friendly to staff, with demonstrable ability to deliver effective feedback in real time that is well received by students. The *Rapid Feedback* application has helped to close the learning loop by delivering feedback to meet the students’ needs. Future work could explore comparing differences between verbal and written feedback in teaching communication skills, the impact of feedback on their clinical performance throughout the course, and the suitability to adapt this application for clinical assessments.

## Take Home Messages

• A digital automated feedback application helps students to reflect on their performance, potentially enhancing personal growth and communication behaviors.
• The application has helped to close the learning loop by delivering feedback to meet the students’ needs.

## Notes On Contributors

Dr Kwang Meng Cham is a lecturer who is primarily involved in preclinical teaching in the early years of student training. He has been successful with several University Learning and Teaching Initiative Grants looking at using technology to enhance formative and summative assessment in Optometry and allied health disciplines.

Dr Jia Jia Lek is a lecturer at the Department of Optometry and Vision Sciences at the University of Melbourne. She has extensive experience in the teaching and learning of Optometry and Opticianry.

Dr Jeremiah Lim is a sessional demonstrator at the Department of Optometry and Vision Sciences at the University of Melbourne. He is also a clinical teaching instructor at the University of Melbourne Eye Care clinic where he supervises students in his capacity as a clinical optometrist.

Ms Anthea Cochrane is a senior lecturer at the Department of Optometry and Vision Sciences at the University of Melbourne. She has been successful with several University Learning and Teaching Grants and is interested in improving assessment and feedback to students.
