# Supplementary material for: Use of an automated feedback application to improve communication skills
Source: MedEdPublish (2016). 2018 Jan 15;7:11. [Version 1] doi: 10.15694/mep.2018.0000011.1 (PMC10699360; doi:10.15694/mep.2018.0000011.1)
Supplement: Peer review history

# Reviewer report: Reviewer response for version 1

**DOI:** 10.21956/mep.19489.r28789

**Published:** 2018-01-24

**Authors:** BALAJI ARUMUGAM

**Affiliations:** 1 TAGORE MEDICAL COLLEGE AND HOSPITAL

This review has been migrated. The reviewer awarded 4 stars out of 5 Good attempt in making and providing the timely feedback to improve the communication skills for the medical students. Congrats to the authors. Clarification on the Rapid Feedback application was not sufficient. But the results of the study was really good.

Reviewer Expertise:

NA

No decision status is available

# Reviewer report: Reviewer response for version 1

**DOI:** 10.21956/mep.19489.r28787

**Published:** 2018-01-17

**Authors:** Tripti Srivastava

**Affiliations:** 1 Datta Meghe Institute of Medical Sciences Deemed University

This review has been migrated. The reviewer awarded 2 stars out of 5 The manuscript strikes a pertinent aspect of learning i.e timely feedback. The authors have used ‘Rapid feedback application’ for giving immediate feedback to the students about voice, pace and confidence, presentation structure, quality of slides/visual aids, knowledge of material, content and concluding remarks of oral presentation. It will be worthwhile to give more details about this feedback method. Student satisfaction regarding feedback is also obtained which also accounts for more transparency. Follow up in subsequent oral presentations can prove to be useful in assessing the overall impact of feedback.

Reviewer Expertise:

NA

No decision status is available

# Reviewer report: Reviewer response for version 1

**DOI:** 10.21956/mep.19489.r28785

**Published:** 2018-01-16

**Authors:** P Ravi Shankar

**Affiliations:** 1 American International Medical University

This review has been migrated. The reviewer awarded 3 stars out of 5 This is a short and interesting manuscript detailing the use of technology to provide feedback to students. While I do agree that presentation skills is a part of the domain of communication skills there may be differences involved when comparing direct communication with patients in a clinical setting to giving a presentation to patients and/or fellow students and faculty. While the nature of the presentation and the audience has not been specified I assume the presentations were to other groups of students. The authors should mention among which group of students the application was tested. The applications ‘Rapid feedback’ seems to be useful but as pointed out by the other reviewers more information may be required.

Reviewer Expertise:

NA

No decision status is available

# Reviewer report: Reviewer response for version 1

**DOI:** 10.21956/mep.19489.r28786

**Published:** 2018-01-15

**Authors:** Mohammed Osman

**Affiliations:** 1 General orgainization for teaching hospitals

This review has been migrated. The reviewer awarded 3 stars out of 5 Interesting maniscript and idea.However, there was no mention of the development of ( The Rapid Feedback application ) including its validity and reliability.The conclusion was extensive and needs to be summerised.

Reviewer Expertise:

NA

No decision status is available

# Reviewer report: Reviewer response for version 1

**DOI:** 10.21956/mep.19489.r28790

**Published:** 2018-01-15

**Authors:** Trevor Gibbs

**Affiliations:** 1 AMEE

This review has been migrated. The reviewer awarded 3 stars out of 5 An interesting paper that approaches an important area of student development. The paper however does raise some conflicts in my understanding of the value of this activity. Effective feedback, as the authors' point out, needs to be timely and the initial feedback post activity addresses this- I was not sure what extra the automated feedback provided over time. Feedback I believe is a two way process so again I would have liked to have seen how the feedback evolved over time. There was short reference made to how the faculty felt- I would have liked to have seen further expansion on this given that to me it promotes extra work to the faculty- work that seems repetitive. I agree that having a written form of feedback does assist the student in their future development. I have little doubt that this is a valuable development, but I feel that greater clarification and more qualitative evaluation is required to make this into a better piece of research

Reviewer Expertise:

NA

No decision status is available

# Reviewer report: Reviewer response for version 1

**DOI:** 10.21956/mep.19489.r28788

**Published:** 2018-01-15

**Authors:** Johnny Lyon Maris

**Affiliations:** 1 Southampton GP Education Unit

This review has been migrated. The reviewer awarded 4 stars out of 5 This is an excellent example of 'timely' feedback. I would like to have known more about the pre-prepared statements, as although they were adapted for each student, there is a possibility of losing their effectiveness if the students share their feedback. It would have been excellent to see the feedback sheets having external evaluation by faculty, sometimes students are biased in their feedback so attaining honest and informed feedback is difficult with early learners.Overall a well set out study, written clearly in a topical and important area.

Reviewer Expertise:

NA

No decision status is available
